# Association between serum calcium and in-hospital mortality in intensive care unit patients with cerebral infarction: a cohort study

**DOI:** 10.3389/fneur.2024.1428868

**Published:** 2024-10-18

**Authors:** Kaiwu Meng, Xiaoyang Lei, Dian He

**Affiliations:** ^1^Neurology Department, Affiliated Hospital of Guizhou Medical University, Guiyang, Guizhou, China; ^2^Neurology Department, The People's Hospital of QianNan, Duyun, Guizhou, China

**Keywords:** serum calcium, cerebral infarction, intensive care unit, in-hospital mortality, nonlinear relationship

## Abstract

**Background:**

The relationship between serum calcium levels and the prognosis of cerebral infarction remains controversial.

**Purpose:**

This study aims to investigate the correlation between serum calcium levels and in-hospital mortality in critically ill patients with ischemic stroke admitted to the intensive care unit (ICU).

**Methods:**

A retrospective cohort study was conducted using data from the MIMIC-IV database. Demographic and clinical data of all participants were collected including gender, age, hypertension, diabetes, myocardial infarction, heart failure, chronic obstructive pulmonary disease, hemoglobin, potassium, sodium, anion gap, platelets, white blood cells, glucose, creatinine, Glasgow coma score (GCS), IV-tPA administration (rt-PA), and mechanical thrombectomy (MT). The outcome measure was in-hospital death. Multivariable-adjusted logistic regression analysis, curve fitting, interaction analysis, and threshold effect analysis were employed to evaluate the relationship between serum calcium levels and in-hospital mortality among ICU patients with cerebral infarction.

**Results:**

A total of 2,680 critically ill patients with cerebral infarction were enrolled, with a mean serum calcium level of 8.6 ± 0.8 mg/dL. The overall in-hospital mortality rate was 19.5%, where Group 1 (serum calcium < 8.0 mg/dL) had a mortality rate of 27.7%, Group 2 (serum calcium 8–9 mg/dL) had a rate of 19.8%, and Group 3 (serum calcium ≥ 9 mg/dL) had a rate of 13.9%. There was a non-linear, S-shaped relationship between serum calcium levels and in-hospital mortality. Serum calcium levels within the range of 7.70–9.50 mg/dL were found to be independently associated with increased in-hospital mortality in ICU patients with cerebral infarction. No significant interactions were detected in subgroup analyses, and the results of sensitivity analyses remained stable.

**Conclusion:**

Serum calcium levels are independently associated with in-hospital mortality in critically ill patients with cerebral infarction in the ICU setting. Within the range of 7.70–9.50 mg/dL, lower serum calcium levels increase the risk of in-hospital death among these patients, emphasizing the importance of close monitoring by ICU physicians.

## 1 Introduction

Cerebral infarction has received worldwide attention due to its high incidence and mortality rates, as well as its potential to cause disability. In recent years, numerous biological markers associated with the prognosis of cerebral infarction have been investigated and confirmed, such as the triglyceride-glucose index and the stress hyperglycemia ratio (1, 2). However, the relationship between serum calcium levels and the prognosis of cerebral infarction remains controversial. In humans, serum calcium levels are maintained within a relatively narrow range through a series of intricate feedback regulatory mechanisms (3); calcium ions play crucial roles in various physiological processes, particularly in the nervous system, where they participate in the conduction of nerve impulses, influence neuronal excitability, and regulate neurotransmitter release, thereby affecting the transmission of neural signals. Prior research has suggested that lower calcium levels may be associated with poorer clinical outcomes in ischemic stroke patients (4–7). Some studies have also linked low serum calcium levels to hemorrhagic transformation following cerebral infarction (8, 9). A study involving 784 patients revealed a nonlinear relationship between serum calcium levels and the prognosis of ischemic stroke, indicating that both excessively low and high calcium concentrations can elevate the likelihood of adverse outcomes (10). Contradictory findings have been reported by other research as well (11–13). The current debate is whether serum calcium levels are positively or negatively associated with the prognosis of cerebral infarction. In light of this ongoing debate, we conducted a study using a large amount of high-quality data from the MIMIC IV database to clarify the association between serum calcium levels and in-hospital mortality in patients with acute stroke and to explore the optimal range of serum calcium concentrations to improve outcomes in this patient population.

## 2 Methods

### 2.1 Data collection

This study used health-related data obtained from the MIMIC-IV (version 2.2) database, a common and large database developed and managed by the Massachusetts Institute of Technology's Laboratory of Computational Physiology. The database contains extensive and high-quality medical records for more than 70,000 patients admitted to the intensive care unit (ICU) at Beth Israel Deaconess Medical Center in Boston, Massachusetts, between 2008 and 2019 (14). Access to this database is granted to those who successfully complete a training program and examination provided by the collaborating institution; one author of this study obtained access with permission, certificated under number 61405960. Given the retrospective nature of this research and the fact that all patients included in the study were extracted from a publicly available database, informed consent was waived, as per the institutional policies governing the use of such de-identified data for research purposes.

### 2.2 Population selection criteria

**Figure 1:**
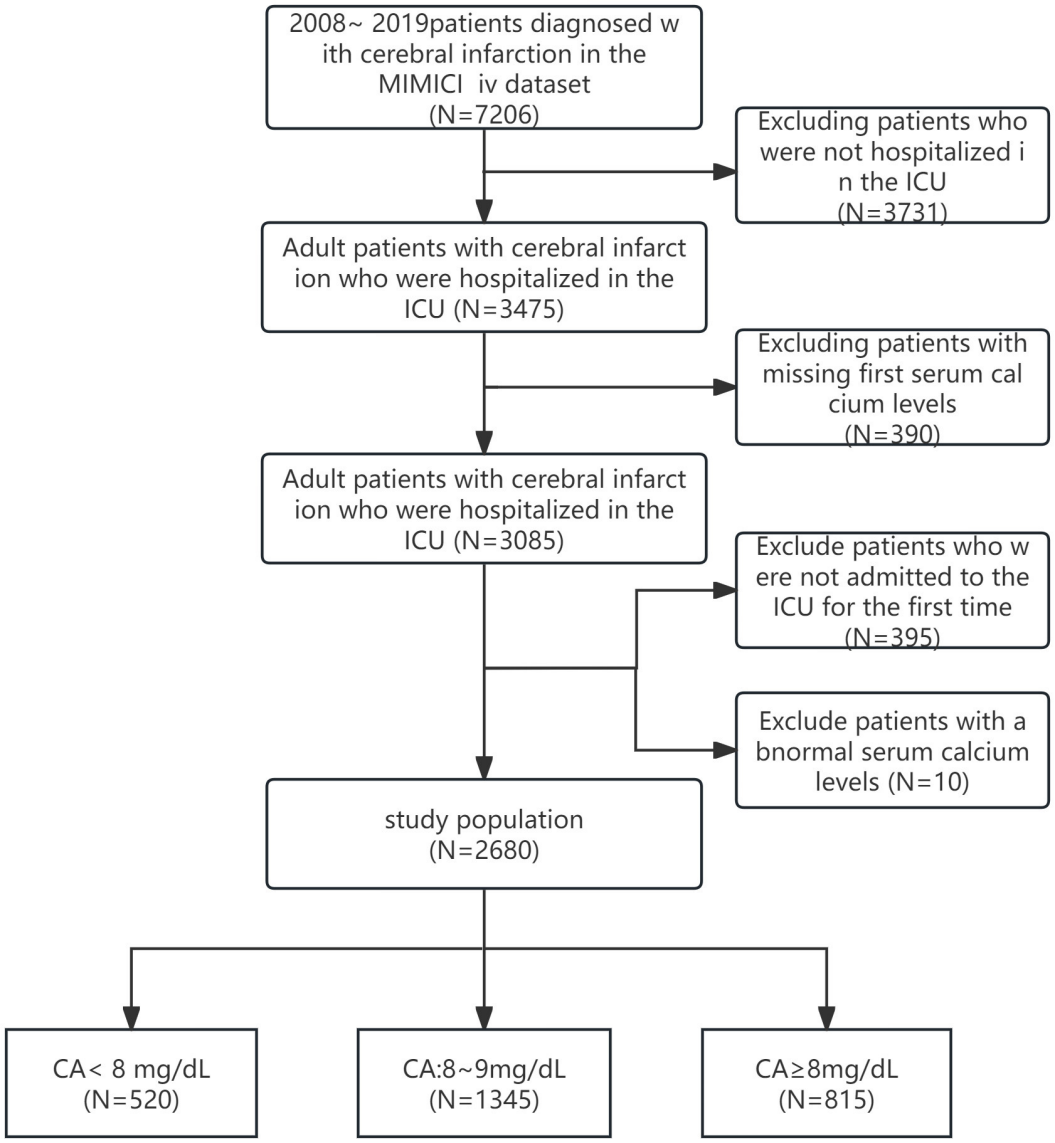
Flowchart of study population. CA, serum calcium.

In the MIMIC-IV (2.2) database, the diagnosis of cerebral infarction was based on the presence of one of the International Classification of Diseases, Ninth Revision (ICD-9) codes 43301, 43311, 43321, 43331, 43381, 43391, 43401, 43411, 43491, or the corresponding ICD-10 code I63. Exclusion criteria were as follows: (1) patients younger than 18 years at the time of the first admission; (2) patients with multiple ICU admissions for cerebral infarction, for which only the first admission data were extracted; (3) patients without ICU admission data; (4) patients with missing serum calcium data or hospital expire flag data on the first day of admission; and (5) the presence of extreme serum calcium values (serum calcium values between 5.4 and 12.1 mg/dL out of range). Out of 7,206 patients extracted from the MIMIC-IV database, 3,731 were excluded due to incomplete ICU admission records, while an additional 390 were excluded because of missing initial serum calcium measurements. Moreover, 395 patients were removed due to multiple ICU stays. Ten more patients were excluded due to serum calcium levels outside the acceptable range (< 5.4 or >12.1 mg/dL). Ultimately, a total of 2,680 ischemic stroke patients fulfilled the eligibility criteria and were included in our study (Figure 1). Information on patient survival was obtained from the 'patients' table within the MIMIC-IV database. The follow-up period was calculated from the date of ICU admission until discharge. Data on the duration of hospitalization were extracted from the “admissions” table within the same database.

### 2.3 Data extraction

In this study, we selected the first recorded serum calcium level following admission as the primary exposure variable of interest. The endpoint of our investigation was in-hospital mortality subsequent to admission to the ICU. Potential confounding factors included age, sex, comorbidities (such as diabetes, hypertension, myocardial infarction, heart failure, chronic pulmonary disease, and GCS); Laboratory examination indicators are selected based on their clinical significance, encompassing the lowest recorded hemoglobin and platelet counts, the highest white blood cell count, creatinine level, glucose level, anion gap, and mean potassium and sodium levels, all measured on the first day in the ICU. Additionally, information regarding whether the patient underwent rt-PA or MT is also included. Data extraction was carried out using PostgreSQL version 13.9 and Navicat Premium version 16.3.3, alongside Structured Query Language (SQL). All codes utilized for computing demographic characteristics, laboratory tests, comorbidities, and severity scores were sourced from the GitHub repository at https://github.com/MIT-LCP/mimic-code.

### 2.4 Management of missing data

In the dataset we collected, there were one missing value for glucose, five missing values for hemoglobin, five missing values for platelets, four missing values for white blood cells, two missing values for anion gap, and five missing values for GCS. The proportion of missing values for all these variables was < 0.2%. To handle these missing data points, we employed a simple imputation method by substituting them with the respective mean values.

### 2.5 Statistical analysis

Utilizing histogram distributions and Q-Q plots, we examined whether variables exhibited normal distribution. Continuous variables conforming to a normal distribution were expressed as means ± standard deviations (mean ± SD), while skewed continuous variables were described using medians [interquartile ranges (IQR)]. Categorical variables were presented as frequencies (%). For baseline characteristic analysis, we utilized chi-square tests for categorical variables, one-way ANOVA for normally distributed continuous variables, and Kruskal-Wallis *H*-tests for non-normally distributed continuous variables to assess differences among distinct groups. Based on serum calcium levels, patients were categorized into three groups: CA < 8 mg/dL, 8 mg/dL ≤ CA < 9 mg/dL, and CA ≥ 9 mg/dL. To investigate the relationship between serum calcium levels and in-hospital mortality following ICU admission, we employed smooth curve fitting and conducted both univariate and multivariate logistic regression analyses. We chose to include adjusted confounders based on their clinical significance and their correlation with the outcome of interest or change in effect estimate beyond 10%. We used unadjusted and multivariable-adjusted models to establish the consistency of the relationship, choosing variables for adjustment if the matched odds ratio altered by at least 10%. In Model 1, adjustments were made for gender and age. Model II built upon Model I by further adjusting for diabetes, hypertension, myocardial infarction, heart failure, and chronic pulmonary disease. Model 3 expanded on Model 2 by adding additional adjustments for hemoglobin, potassium, sodium, anion gap, platelets, white blood cells, creatinine, glucose, GCS, IV-tPA administration, and mechanical thrombectomy. The results were expressed as odds ratios (OR) with 95% confidence intervals (CI). A threshold effect analysis was conducted to evaluate the predictive capacity of serum calcium levels for in-hospital mortality. All comparisons were planned, tests were two-tailed, and a *P*-value < 0.05 signified statistical significance between two or more groups. All analyses were conducted using R version 4.3.2(http://www.R-project.org, R Foundation) and Free Statistics (1.9.2).

## 3 Results

### 3.1 Baseline demographic and clinical characteristics

Following the application of inclusion and exclusion criteria and the elimination of extreme serum calcium values, a total of 2,680 ischemic stroke patients admitted to the ICU for the first time were identified in the MIMIC-IV database (Figure 1). Table 1 presents the basic demographic characteristics of participants stratified by serum calcium levels. The average age of participants was 69.3 ± 15.8 years, with 1,311 individuals (48.9%) being male. The mean initial serum calcium level upon ICU admission was 8.6 ± 0.8 mg/dL. Serum calcium, hemoglobin, anion gap, serum potassium, serum sodium, and GCS had a normal distribution, and platelets, WBC, creatinine, and glucose had a non-normal distribution. There were statistically significant differences in the distribution of potential confounding factors, such as hemoglobin, anion gap, sodium, platelets, white blood cells, glucose, creatinine, and Glasgow coma score, across different serum calcium levels. Among these patients, 46 underwent mechanical thrombectomy procedures, while 49 received intravenous thrombolysis treatment using alteplase.

**Table 1:** Baseline demographic characteristics of the study population stratified by serum calcium.

**Variables**	**Total (*n* = 2,680)**	**Serum calcium (mg/dL)**			***p***
		** < 8 (*n* = 520)**	**8–9 (*n* = 1,345)**	**≥9 (*n* = 815)**	
Sex, *n* (%)					0.103
Female	1,369 (51.1)	250 (48.1)	680 (50.6)	439 (53.9)	
Male	1,311 (48.9)	270 (51.9)	665 (49.4)	376 (46.1)	
Age, (year)	69.3 ± 15.8	66.8 ± 16.4	69.9 ± 15.5	70.0 ± 15.8	< 0.001
Myocardial infarct, *n* (%)					0.001
No	2,231 (83.2)	411 (79)	1,114 (82.8)	706 (86.6)	
Yes	449 (16.8)	109 (21)	231 (17.2)	109 (13.4)	
Heart failure, *n* (%)					0.021
No	1,969 (73.5)	362 (69.6)	984 (73.2)	623 (76.4)	
Yes	711 (26.5)	158 (30.4)	361 (26.8)	192 (23.6)	
Chronic pulmonary disease, *n* (%)					0.002
No	2,164 (80.7)	401 (77.1)	1,075 (79.9)	688 (84.4)	
Yes	516 (19.3)	119 (22.9)	270 (20.1)	127 (15.6)	
Diabetes, *n* (%)					0.06
No	1,809 (67.5)	355 (68.3)	930 (69.1)	524 (64.3)	
Yes	871 (32.5)	165 (31.7)	415 (30.9)	291 (35.7)	
Hypertension, *n* (%)					0.002
No	2,022 (75.4)	409 (78.7)	1,033 (76.8)	580 (71.2)	
Yes	658 (24.6)	111 (21.3)	312 (23.2)	235 (28.8)	
Calcium (mg/dL)	8.6 ± 0.8	7.4 ± 0.5	8.5 ± 0.3	9.4 ± 0.4	< 0.001
Hemoglobin (g/dL)	11.2 ± 2.4	9.8 ± 2.3	11.1 ± 2.2	12.1 ± 2.2	< 0.001
Anion gap (mmol/L)	16.4 ± 4.3	17.1 ± 5.4	16.1 ± 4.1	16.6 ± 3.9	< 0.001
Potassium (mmol/L)	4.1 ± 0.6	4.2 ± 0.6	4.1 ± 0.6	4.1 ± 0.6	0.881
Sodium (mmol/L)	139.4 ± 4.3	138.7 ± 5.0	139.3 ± 4.1	139.9 ± 4.1	< 0.001
rt_PA, *n* (%)					0.083
No	2,631 (98.2)	510 (98.1)	1,314 (97.7)	807 (99)	
Yes	49 (1.8)	10 (1.9)	31 (2.3)	8 (1)	
MT, *n* (%)					0.016
No	2,634 (98.3)	518 (99.6)	1,314 (97.7)	802 (98.4)	
Yes	46 (1.7)	2 (0.4)	31 (2.3)	13 (1.6)	
GCS	10.8 ± 3.8	9.8 ± 4.1	10.8 ± 3.8	11.5 ± 3.6	< 0.001
Platelet count (× 10^9^/*L*)	197.0 (149.0, 253.0)	164.0 (106.5, 221.5)	197.0 (150.0, 254.0)	212.0 (171.0, 263.8)	< 0.001
WBC (× 10^9^/*L*)	11.6 (8.7, 15.6)	13.7 (9.9, 18.4)	11.4 (8.7, 15.1)	10.7 (8.4, 14.2)	< 0.001
Creatinine (mg/dL)	1.0 (0.8, 1.4)	1.2 (0.8, 2.0)	1.0 (0.8, 1.3)	1.0 (0.8, 1.3)	< 0.001
Glucose (mg/dL)	139.0 (111.0, 186.0)	152.0 (122.0, 210.0)	136.0 (109.0, 178.0)	135.0 (110.0, 183.0)	< 0.001

Data presented are mean ± SD, median (IQR), or *N* (%).SD, standard deviation; IQR, interquartile range; WBC, white blood cell; GCS, Glasgow coma score; rt-PA, intravenous thrombolysis; MT, mechanical thrombectomy.

### 3.2 Serum calcium is an independent risk factor for in-hospital mortality

After univariate analysis, age, hypertension, myocardial infarction, heart failure, chronic pulmonary disease, hemoglobin, platelets, white blood cells, potassium, anion gap, creatinine, glucose and GCS were found to be significantly related to in-hospital mortality. Conversely, sex, diabetes, sodium, rt-PA, and MT did not show significant associations with in-hospital mortality. In the multivariable logistic regression analysis (Table 2), when serum calcium was treated as a continuous variable, lower serum calcium levels were associated with an increased risk of in-hospital death in ICU patients with cerebral infarction (OR = 0.69, 95% CI: 0.61–0.77, *P* < 0.001). This conclusion remained consistent even after adjusting for confounding variables including sex, age, diabetes, hypertension, myocardial infarction, congestive heart failure, chronic pulmonary disease, hemoglobin, platelets, anion gap, white blood cells, glucose, creatinine, potassium, sodium, GCS, rt-PA, and MT (Model 3, OR = 0.84, 95% CI: 0.73–0.97, *P* = 0.015). When serum calcium was considered a categorical variable, the third group (CA ≥ 9 mg/dL) served as the reference category. The first group (CA < 8 mg/dL) had an OR of 2.38 (95% CI: 1.8–3.14, *P* < 0.001) and the second group (8 ≤ CA < 9 mg/dL) had an OR of 1.53 (95% CI: 1.21–1.95, *P* < 0.001), both demonstrating a higher risk of in-hospital mortality. The OR for serum calcium levels remained stable across the three models after adjustments in the multivariate model.

**Table 2:** Relationship between different serum calcium levels and in-hospital morality in different models.

**Variable**	***n*.total**	***n*.event_%**	**Unadjusted**		**Model 1**		**Model 2**		**Model 3**	
			**OR (95% CI)**	***P***	**OR (95% CI)**	**P**	**OR (95% CI)**	**P**	**OR (95% CI)**	**P**
Calcium	2,680	523 (19.5)	0.69 (0.61–0.77)	< 0.001	0.66 (0.58–0.75)	< 0.001	0.68 (0.6–0.77)	< 0.001	0.84 (0.73–0.97)	0.015
CA < 8 (mg/dL)	520	144 (27.7)	2.38 (1.8–3.14)	< 0.001	2.55 (1.93–3.37)	< 0.001	2.43 (1.84–3.23)	< 0.001	1.54 (1.11–2.13)	< 0.009
8 ≤ CA < 9 (mg/dL)	1,345	266 (19.8)	1.53 (1.21–1.95)	< 0.001	1.54 (1.21–1.96)	< 0.001	1.52 (1.18–1.91)	0.001	1.33 (1.02–1.73)	0.037
CA ≥ 9 (mg/dL)	815	113 (13.9)	1 (Ref)		1 (Ref)		1 (Ref)		1 (Ref)	

Data presented are ORs and 95% CIs. Model 1: adjusted for age, sex; Model 2: adjusted for age, sex, heart failure, hypertension, diabetes, myocardial infarct, chronic pulmonary disease; Model 3: adjusted for age, sex, heart failure, hypertension, diabetes, myocardial infarct, Chronic pulmonary disease, hemonglobin, potassium, sodium, anion gap, plateles, GCS, WBC, glucose, MT, rt-PA.

### 3.3 The nonlinear relationship and sensitivity analysis

**Figure 2:**
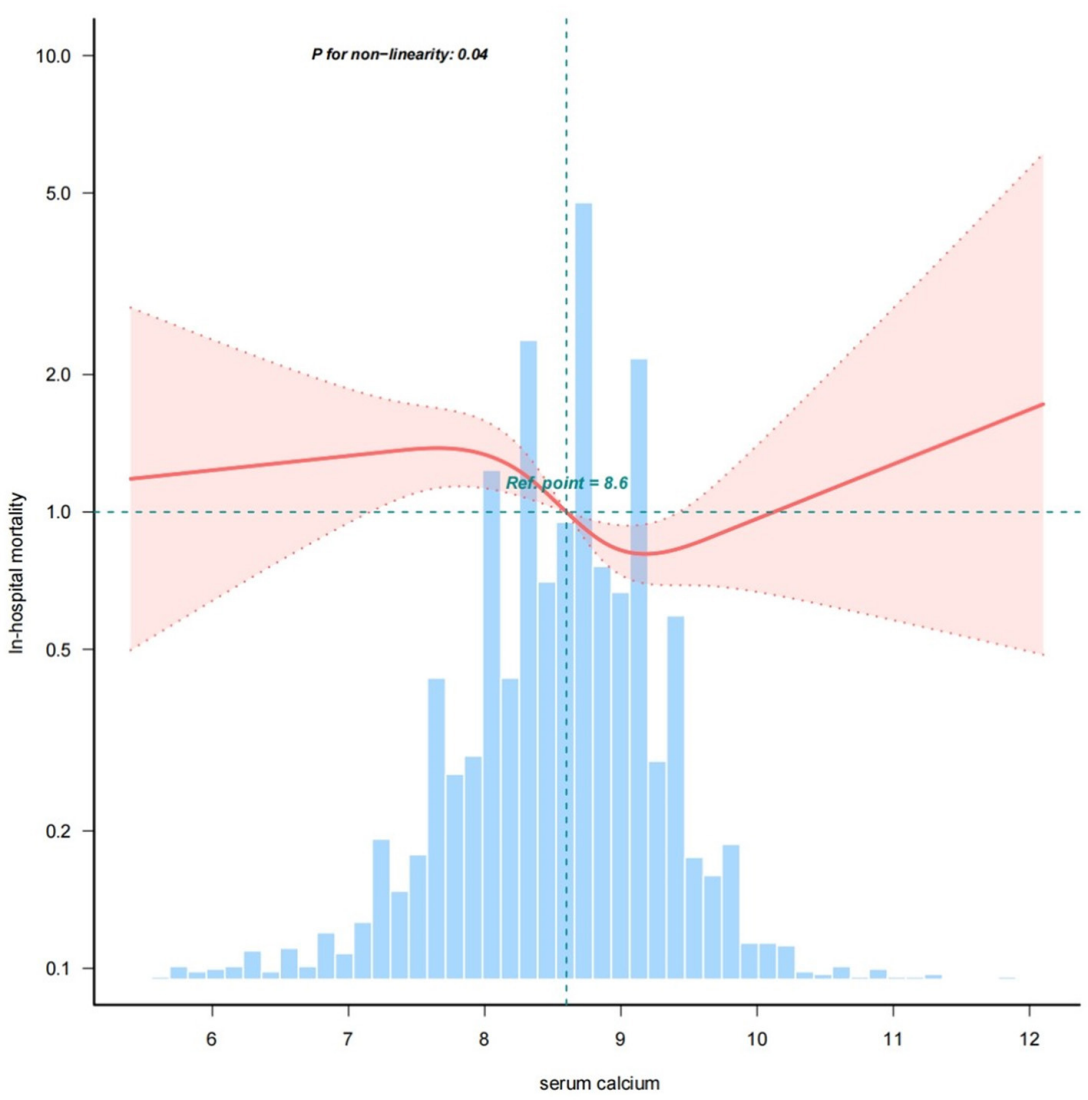
The nonlinear relationship between serum calcium and in-hospital mortality in ICU patients with cerebral infarction (adjusted for all covariates as model 3).

Upon conducting multifactorial logistic regression analysis and spline curve fitting, we uncovered a nonlinear relationship between serum calcium levels and in-hospital mortality within the ICU, with a nonlinearity *P*-value of 0.04 (Figure 2). Following adjustment for confounding variables including sex, age, diabetes, hypertension, myocardial infarction, heart failure, chronic pulmonary disease, hemoglobin, platelets, anion gap, WBC, glucose, creatinine, potassium, sodium, GCS, rt-PA, and MT, no significant correlation was found between in-hospital mortality and changes in serum calcium levels below 7.70 mg/dL (OR = 1.10, 95% CI: 0.62–1.96, *P* = 0.73). However, when serum calcium levels were above 7.70 mg/dL but < 9.50 mg/dL, a decrease of one unit in serum calcium was associated with a 30% reduction in the risk of in-hospital death (OR = 0.70, 95% CI: 0.54–0.92, *p* = 0.01). In a similar vein, at serum calcium concentrations equal to or surpassing the second inflection point value of 9.50 mg/dL, no appreciable relationship was observed between variations in serum calcium and in-hospital mortality (OR = 1.20, 95% CI: 0.43–3.39, *p* = 0.72). We performed logistic multivariable analysis on the three datasets obtained by multiple imputation and found the results to be stable (Supplementary Table 2).

### 3.4 Subgroup analysis and forest plots

**Figure 3:**
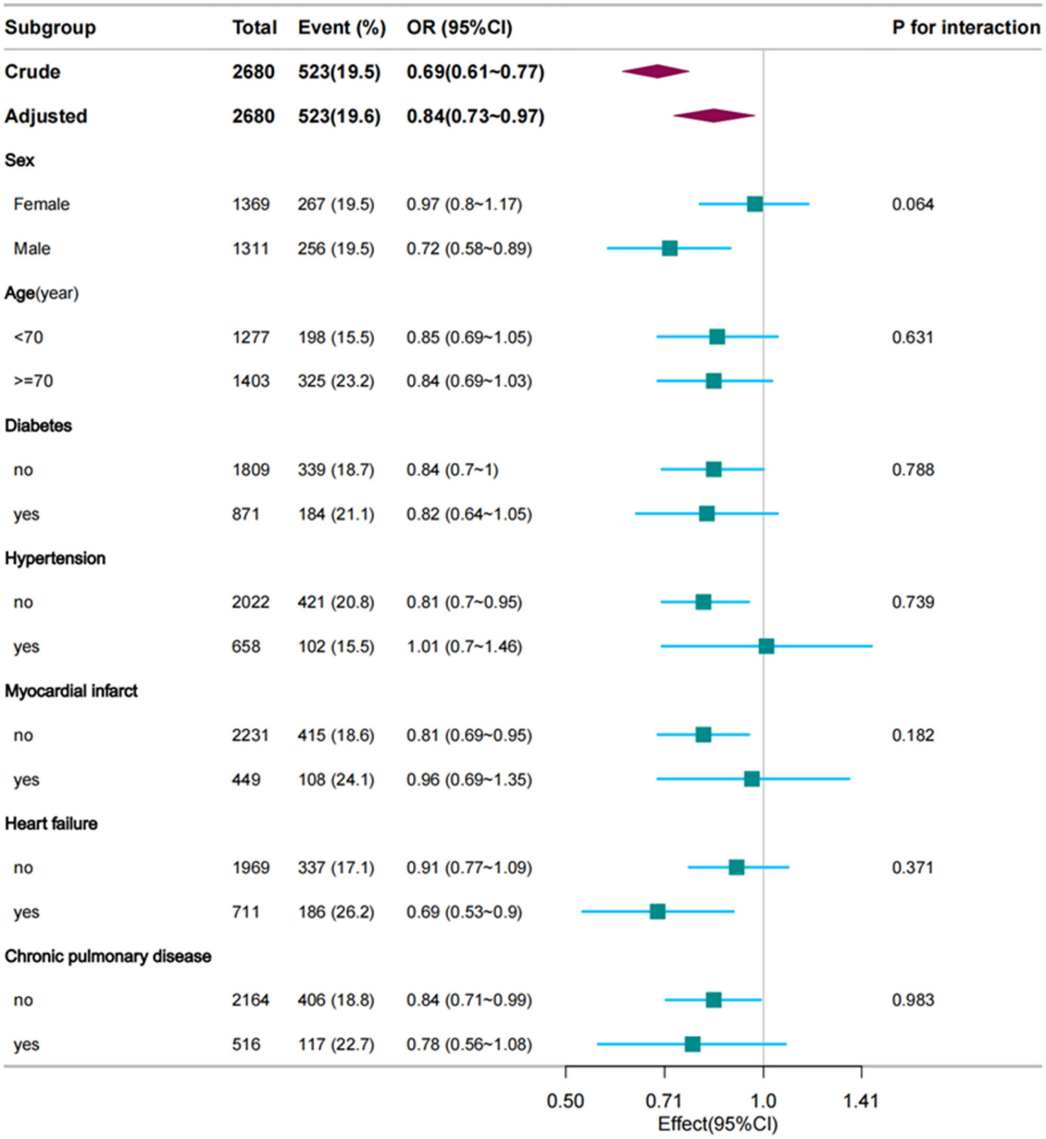
Forest plot of multivariate logistic regression subgroup analysis (adjusted for all covariates as model 3).

The logistic subgroup analysis revealed a significant correlation between decreased serum calcium concentrations and heightened in-hospital mortality among ischemic stroke patients admitted to the ICU. Notably, this correlation was exacerbated, with statistically significant disparities surfacing more prominently in male patients, those devoid of hypertension, as well as individuals concurrently afflicted with heart failure or chronic pulmonary disorders. Analysis did not unveil any statistically meaningful interaction effects across the subgroups (*p* for interaction > 0.05), thereby implying an absence of considerable modulatory impacts from demographic or clinical variables on the link between reduced calcium levels and in-hospital death rates (Figure 3).

## 4 Discussion

In this retrospective cohort study utilizing MIMIC IV data, we distinctly demonstrated that lower serum calcium levels are independently associated with an increased risk of in-hospital mortality. This relationship manifests as a nonlinear pattern (nonlinear *P*-value < 0.05), wherein each decrement of 1 unit in serum calcium within the range of 7.70–9.50 mg/dL corresponded to a 30% heightened risk of inpatient death. Subsequent subgroup analyses revealed analogous patterns of association. These findings bear significant clinical implications.

Evidence continues to emerge on the relationship between serum calcium levels and prognosis in stroke patients, but the results are inconsistent. Previous systematic reviews have found an association between serum calcium levels and mortality from cerebrovascular disease, but have not elucidated the nature of this association (15). The results of a meta-analysis showed that low serum calcium levels are associated with hemorrhagic transformation after cerebral infarction (16). There are also retrospective studies showing that serum calcium levels are not associated with the prognosis of cerebral infarction (17). The main controversy at present is whether serum calcium levels are positively or negatively correlated with cerebral infarction. A number of studies have reported an increase in the severity of cerebral infarction with increasing serum calcium levels, and a positive correlation between infarct size and elevated calcium levels has been observed (11–13). Our study confirms the independent association between serum calcium level and in-hospital mortality in patients with cerebral infarction and shows an “s”-shaped nonlinear relationship. Lower serum calcium levels between 7.70 and 9.5 mg/dL were associated with an increased risk of in-hospital mortality, and there was a trend toward decreased mortality below 7.7 mg/dL, but this was not statistically significant, which may be due to bias caused by the small number of patients below 7.7 mg/dL. In contrast, a trend toward an increased risk of mortality was observed at serum calcium levels > 9.5 mg/dL, but again, this was not statistically significant. Our results suggest that patients with cerebral infarction who have lower serum calcium levels are at a higher risk of in-hospital mortality. Whether an increase in serum calcium concentration at serum calcium levels below 9.50 mg/dL is beneficial in improving in-hospital survival in patients with cerebral infarction requires further investigation.

These findings are consistent across subgroups defined by different ages, genders, races, and the presence or absence of comorbid conditions like diabetes and hypertension. The underlying mechanisms through which serum calcium levels influence the prognosis of cerebral infarction patients remain to be elucidated. Conventionally, calcium has been thought to play a detrimental role in the pathogenesis of stroke; however, trials employing calcium channel blockers for the treatment of cerebral infarction have not yielded positive outcomes (18). The likely mechanism is that under ischemia and hypoxia, glutamate is released from neurons and glial cells, which activates N-methyl-D-aspartate (NMDA) receptors, leading to an influx of calcium ions into the cell. This massive influx of calcium triggers the activation of lethal second messengers and enzymes, mitochondrial dysfunction, inflammatory cellular infiltration, and increased production of free radicals. Together, these events lead to neuronal cell death and brain damage, promoting a deleterious feedback loop of further calcium ion influx (19). The results of our study are consistent with observations from basic studies suggesting an association between reduced serum calcium levels and increased risk of death in patients with cerebral infarction.

In the context of substantial debate over the relationship between serum calcium levels and stroke prognosis, we assessed the association between serum calcium ions and in-hospital mortality rates among patients with cerebral infarction in the ICU. This study represents a large-sample retrospective cohort study, offering sufficient statistical power to support the majority of subgroup analyses. Additionally, we excluded 10 patients whose initial serum calcium measurement upon ICU admission was either below 5.4 mg/dL or above 12.1 mg/dL, recognizing that such extreme values could potentially impact our findings. This real-world study yields objective insights into the relationship between serum calcium levels and mortality rates for ICU patients with cerebral infarction, adopting a novel and clinically relevant approach with high practical application value.

Our study provides strong evidence of the association between serum calcium concentration and in-hospital mortality in ischemic stroke patients admitted to the intensive care unit by rigorously reducing a number of potential confounders and sources of bias. First, the diagnostic dependence on ICD codes introduces the possibility of misclassification, even when important diagnostic categories are used. Second, our study focused only on the ICU cohort, which minimally included patients who underwent intravenous thrombolysis or mechanical thrombolysis, which may affect the generalizability of our findings, and new studies are needed to increase the generalizability of the results of the present study; third, data on prehospital medications were not documented in the MIMIC database, so that the impact of this confounding factor on the present study could not be assessed in our study; fourth, the lack of NIHSS score data in the MIMIC IV database hindered its use as a covariate indicating neurological impairment, but we included GCS as a partial compensatory measure for this deficiency. In addition, we used mean substitution for missing values, which is not sufficiently scientific, but given that the missing data were random and < 0.2% of the data were missing, their impact on our results was not significant. This was confirmed by the results of logistic multifactorial analysis after multiple interpolation of missing values. Finally, given that previous studies have failed to demonstrate an association between serum albumin concentration, atrial fibrillation, and in-hospital mortality in patients (20, 21), we did not include these confounding factors into the adjustment model.

Despite certain limitations, our study comprehensively investigated the association between serum calcium levels and in-hospital mortality in patients with cerebral infarction in the ICU, thus contributing to the body of evidence on this topic. Our conclusions emphasize that our study demonstrated an independent effect of serum calcium levels on in-hospital mortality after accounting for confounding factors. In this study, we observed an “S”-shaped nonlinear relationship between calcium levels and in-hospital mortality, particularly in the range of 7.70–9.50 mg/dL, where lower calcium levels were associated with a higher risk of death. The findings of the present study are noteworthy. Considering that existing studies have shown that increasing calcium intake can reduce the incidence of stroke (22–24), and taking into account the results of our study, the effect of increasing serum calcium levels within a reasonable range on the prognosis of cerebral infarction is a worthwhile direction for further research.

## 5 Conclusion

By analyzing data from 2,680 patients with acute cerebral infarction in the MIMIC IV database, we identified a significant association between serum calcium levels and the risk of in-hospital mortality in ICU patients with cerebral infarction, independent of confounding factors. A nonlinear relationship was observed, in which lower serum calcium levels were associated with a higher risk of in-hospital mortality within the range of 7.70–9.50 mg/dL. Beyond 9.50 mg/dL, there was an upward trend in mortality risk. The results of this study suggest that ICU physicians should pay more attention to serum calcium levels in patients with cerebral infarction, especially those with lower levels.

## Data Availability

The original contributions presented in the study are included in the article/Supplementary material, further inquiries can be directed to the corresponding author.
