# Supplementary material for: Association between serum calcium and in-hospital mortality in intensive care unit patients with cerebral infarction: a cohort study
Source: Front Neurol. 2024 Oct 18;15:1428868. doi: 10.3389/fneur.2024.1428868 (PMC11527662; doi:10.3389/fneur.2024.1428868)
Supplement: Supplementary file 1 [file Table_1.DOCX]

**Supplementary Table1. Association of covariates and in-hospital morality in patients with cerebral infarction.**

| Variable | OR_95CI | P_value |
| --- | --- | --- |
| Calcium | 0.69 (0.61~0.77) | <**0.001** |
| Gender=male | 1 (0.83~1.21) | 0.988 |
| Age | 1.02 (1.01~1.02) | <**0.001** |
| Myocardial infarct=yes | 1.39 (1.09~1.76) | **0.008** |
| Heart failure=yes | 1.72 (1.4~2.1) | <**0.001** |
| Chronic pulmonary disease=yes | 1.27 (1.01~1.6) | **0.044** |
| Diabetes=yes | 1.16 (0.95~1.42) | 0.145 |
| Hypertension=yes | 0.7 (0.55~0.88) | **0.003** |
| Hemoglobin | 0.86 (0.83~0.9) | **<0.001** |
| Platelet count | 1 (1~1) | **<0.001** |
| WBC | 1.04 (1.03~1.06) | **<0.001** |
| Anion gap | 1.1 (1.08~1.12) | **<0.001** |
| Glucose | 1 (1~1) | **<0.001** |
| Creatinine | 1.06 (1.01~1.11) | **0.023** |
| Potassium | 1.39 (1.18~1.62) | **<0.001** |
| Sodium | 1.02 (1~1.04) | 0.086 |
| rt-PA=yes | 1.5 (0.79~2.85) | 0.214 |
| MT=yes | 0.39 (0.14~1.09) | 0.072 |
| GCS | 0.82 (0.8~0.84) | **<0.001** |
