# Supplementary material for: Association between serum calcium and in-hospital mortality in intensive care unit patients with cerebral infarction: a cohort study
Source: Front Neurol. 2024 Oct 18;15:1428868. doi: 10.3389/fneur.2024.1428868 (PMC11527662; doi:10.3389/fneur.2024.1428868)
Supplement: Supplementary file 2 [file Table_2.DOCX]

**Supplementary Table 2. Logistic multifactor analysis with 3 datasets interpolated.**

| Variable |  | n.total | n.event_% |  |  | | unadjusted | | | |  | Model 3 | | |  |
| --- | --- | --- | --- | --- | --- | --- | --- | --- | --- | --- | --- | --- | --- | --- | --- |
|  |  |  |  |  |  | | OR (95%CI) |  | | *P* |  | OR (95%CI) |  | *P* |  |
| calcium |  | 8040 | 1569 (19.5) | | | 0.69 (0.64~0.74) | | | <0.001 | | 0.84 (0.78~0.91) | | | <0.001 | |

Notes:adjusted for age, sex, heart failure, hypertension, diabetes, myocardial infarct, Chronic pulmonary disease, hemonglobin, potassium, sodium, anion gap, plateles, GCS, WBC, glucose, MT, rt-PA.
